# Comprehensive Assessment of Autonomic Nervous System Profiles in Postural Orthostatic Tachycardia Syndrome Among Syncope, Chronic Fatigue, and Post-COVID-19 Patients

**DOI:** 10.3390/diagnostics15222824

**Published:** 2025-11-07

**Authors:** Branislav Milovanovic, Nikola Markovic, Masa Petrovic, Vasko Zugic, Milijana Ostojic, Milovan Bojic

**Affiliations:** 1 Institute for Cardiovascular Diseases “Dedinje”, 11000 Belgrade, Serbia; 2 Faculty of Medicine, University of Belgrade, 11000 Belgrade, Serbia

**Keywords:** neurocardiology, Postural orthostatic tachycardia syndrome (POTS), autonomic nervous system, Chronic Fatigue Syndrome, Post-COVID

## Abstract

**Background/Objectives**: Postural orthostatic tachycardia syndrome (POTS) is a form of dysautonomia characterized by excessive tachycardia during orthostatic stress. It is frequently observed in patients with syncope, Chronic Fatigue Syndrome (CFS), and post-COVID-19 syndrome (PCS), yet the underlying mechanisms may differ across these conditions. This study aimed to assess autonomic nervous system (ANS) function in patients with syncope, CFS of insidious onset, and CFS post-COVID-19 who presented with POTS, and to compare them with age- and sex-matched patients without POTS. **Methods**: In this retrospective cross-sectional study, 138 patients over 18 years of age were included following head-up tilt testing (HUTT). Patients were divided into six groups: syncope with and without POTS, CFS with insidious onset with and without POTS, and CFS post-COVID-19 with and without POTS. All participants underwent HUTT, cardiovascular reflex testing (CART) by Ewing, five-minute resting ECG with short-term Heart Rate Variability (HRV) analysis, and 24 h Holter ECG monitoring. **Results**: The prevalence of POTS across groups ranged from 5% to 7%. Female predominance was consistent across all subgroups. In syncope with POTS, hypertensive responses during HUTT, lower rates of normal Valsalva maneuver results, and reduced HF values in short-term HRV suggested baroreceptor dysfunction with sympathetic overdrive. In both CFS subgroups with POTS, CART revealed higher rates of definite parasympathetic dysfunction, along with more frequent extreme blood pressure variation during HUTT and reduced vagally mediated HRV parameters (rMSSD, pNN50). Across groups, no significant differences were observed with regard to long-term HRV across groups. **Conclusions**: Distinct autonomic profiles were identified in POTS patients depending on the underlying condition. Syncope-related POTS was associated with baroreceptor dysfunction and sympathetic predominance, whereas CFS-related POTS was characterized by parasympathetic impairment and impaired short-term baroreflex regulation. Evaluating dysautonomia patterns across disease contexts may inform tailored therapeutic strategies and improve management of patients with POTS.

## 1. Introduction

Postural Orthostatic Tachycardia Syndrome (POTS) is one of the primary clinical manifestations of dysautonomia, characterized by an excessive increase in heart rate upon assuming an upright posture [1]. The hallmark of POTS is an increase in heart rate exceeding 30 beats per minute, or reaching more than 120 beats per minute within 10 min of standing, in the absence of Orthostatic Hypotension (OH). This is accompanied by symptoms of orthostatic intolerance such as syncope, dizziness, and blurred vision, after excluding other potential causes of tachycardia (e.g., anxiety, inappropriate sinus tachycardia, anemia, myocarditis, metabolic disorders). Symptoms must persist for more than six months to establish the diagnosis [1]. Alongside classic OH, initial and delayed OH, and vasovagal syncope (VVS), POTS is recognized by the European Society of Cardiology (ESC) as one of the principal pathophysiological mechanisms underlying orthostatic intolerance [2]. Although the pathophysiology of POTS is not yet fully elucidated, several mechanisms have been proposed, giving rise to different subtypes, including neuropathic POTS, hyperadrenergic POTS, norepinephrine transporter (NET) deficiency, and hypovolemic POTS [3].

Syncope, characterized by a transient loss of consciousness resulting from cerebral hypoperfusion, occurs in approximately 20% of patients with POTS [4]. However, it is generally considered an uncommon manifestation in this population, suggesting that syncope may rarely be a direct consequence of POTS [4]. Nonetheless, both conditions share prodromal symptoms such as dizziness, faintness, and blurred vision, which may occur during POTS episodes or precede syncope.

Post-COVID-19 syndrome (PCS) has emerged as a major sequela of acute SARS-CoV-2 infection, increasingly capturing attention within the medical community [5]. In parallel, Chronic Fatigue Syndrome (CFS) is a debilitating multisystem disorder whose prevalence has been shown to rise following PCS, with an estimated 13–20% of PCS patients fulfilling diagnostic criteria for CFS [6]. A central feature common to both PCS and CFS is orthostatic intolerance, frequently attributable to POTS. Reported prevalence of POTS in CFS ranges from 5.7% to 70%, largely depending on diagnostic criteria and study populations, while prevalence in PCS has been reported between 2% and 14% [7,8,9].

A unifying aspect of these conditions is autonomic nervous system (ANS) dysfunction. In POTS, mechanisms such as a hyperadrenergic response or partial sympathetic neuropathy affecting peripheral vasculature have been implicated [3]. In CFS, the identification of specific patterns of autonomic dysfunction is thought to be essential for determining disease severity and prognosis [10]. In PCS, SARS-CoV-2 has been proposed to damage the ANS through retrograde axonal transport, cytokine storm, systemic inflammation, and autoimmune mechanisms [11]. Another shared characteristic across these disorders is the predominance of female patients and the frequent onset following a viral-like illness [3,12,13,14,15,16,17].

The aim of this study was to evaluate autonomic nervous system function in patients with syncope, Chronic Fatigue Syndrome with insidious onset, and Chronic Fatigue Syndrome following COVID-19 who presented with POTS during head-up tilt testing. Results were compared with age- and sex-matched patients with the aforementioned conditions who did not develop POTS during tilt testing.

## 2. Materials and Methods

### 2.1. Study Protocol

This retrospective, cross-sectional study included 138 patients over the age of 18 who were evaluated at the Neurocardiological Laboratory of the Cardiology Clinic, Institute for Cardiovascular Diseases “Dedinje”, from January 2023 to November 2024. Initially, 1055 patients were screened and categorized into three groups: 287 with Chronic Fatigue Syndrome (CFS) following COVID-19, 501 with CFS of insidious onset, and 267 with syncope. After head-up tilt testing (HUTT), 22 patients (7.7% of 287) with post-COVID-19 CFS, 27 patients (5.4% of 501) with CFS of insidious onset, and 20 patients (7.5% of 267) with syncope fulfilled diagnostic criteria for POTS. Each subgroup of patients who fulfilled diagnostic criteria for POTS (22 post-COVID-19 CFS, 27 CFS of insidious onset, and 20 with syncope) was matched by age and sex with an equal number of patients with the same underlying condition, who did not develop POTS during head-up tilt testing, serving as the control groups. In the CFS groups, patients were additionally required to have symptoms of orthostatic intolerance lasting longer than six months.

POTS was defined as an increase in heart rate of more than 30 beats per minute or a rate exceeding 120 beats per minute during the first 10 min of the passive phase of HUTT, without evidence of OH within 3 min of tilt initiation, accompanied by symptoms of orthostatic intolerance lasting longer than six months [3]. Exclusion criteria for POTS groups included the presence of overt causes of sinus tachycardia, such as acute physiological stimuli, dietary factors, other medical conditions, or medication use [3].

CFS was diagnosed according to the National Institute for Health and Care Excellence (NICE) 2021 guidelines [18], which include the following: (1) debilitating fatigue worsened by activity, not attributable to excessive exertion, and not relieved by rest; (2) post-exertional malaise; (3) unrefreshing sleep or sleep disturbance (including flu-like fatigue, stiffness upon waking, fragmented or shallow sleep, altered circadian rhythm, or hypersomnia); and (4) cognitive impairment (“brain fog”). Exclusion criteria included other medical or psychiatric conditions (e.g., endocrine/metabolic disorders, cardiovascular or neurological disease, primary anxiety or depression, sleep apnea). For patients with post-COVID-19 CFS, symptom onset had to occur after recovery from acute SARS-CoV-2 infection, confirmed according to WHO guidelines by real-time reverse transcription polymerase chain reaction (RT-PCR) using a SARS-CoV-2 nucleic acid detection kit following the manufacturer’s protocol [19]. For patients with post-COVID-19 CFS, symptom onset had to occur after recovery from acute SARS-CoV-2 infection. Recovery was defined according to WHO criteria as clinical resolution of acute illness and at least one negative RT-PCR test result obtained ≥ 10 days after the initial positive test, or the passage of ≥14 days in asymptomatic or mild cases without residual symptoms [19].

Syncope was defined as transient loss of consciousness (TLOC) due to cerebral hypoperfusion, characterized by rapid onset, short duration, and spontaneous recovery [2]. Exclusion criteria for the syncope groups included cardiogenic causes, epilepsy (confirmed by a neurologist), Orthostatic Hypotension secondary to volume depletion or drug use, and primary autonomic neuropathies (e.g., Parkinson’s disease, pure autonomic failure, multiple system atrophy).

All patients underwent HUTT, cardiovascular reflex testing (Ewing battery), 5 min resting ECG recordings in the supine position, and 24 h Holter ECG monitoring.

The study was conducted in accordance with the Declaration of Helsinki and approved by the Institutional Ethics Committee of the Institute for Cardiovascular Diseases “Dedinje” (protocol code 6472; approval date 11 December 2024). This work was supported by the Ministry of Education, Science and Technological Development of the Republic of Serbia (grant 451-03-68/2020-14/200156) and by the Science Fund of the Republic of Serbia (“Assessment of autonomic nervous system dysfunction in patients with COVID-19 infection, analytically supported by artificial intelligence” (COVANSA) grant).

### 2.2. Cardiovascular Reflex Tests by Ewing (CART)

The Ewing cardiovascular reflex test battery was used to assess autonomic function, comprising five tests [20]. Sympathetic function was evaluated with the Hand grip test (HGT) and the blood pressure response to standing test (OH). Parasympathetic function was assessed with the Valsalva maneuver (VM), heart rate response to deep breathing (HRB), and heart rate response to standing (HRT).

According to Ewing’s criteria, results were classified as normal, borderline, or abnormal [20]. Each result was assigned a numerical value: 0 for normal, 1 for borderline, and 2 for abnormal [21]. Scores were summed to yield a total CART score ranging from 0 to 10. Sympathetic dysfunction was defined as at least one abnormal sympathetic test. Parasympathetic dysfunction was classified as “initial” if one test was abnormal and “definite” if two or more tests were abnormal [20].

### 2.3. Head-Up Tilt Test (HUTT)

The Westminster protocol was applied [22]. Patients rested supine for 10 min before tilting. The tilt angle was 70°, and the passive phase lasted up to 30 min. The test was considered positive in the presence of syncope or severe presyncope, accompanied by a blood pressure drop or bradycardia. Blood pressure and a 12-lead ECG were continuously monitored.

Additional hemodynamic changes recorded during HUTT included the following:

1. Extreme variation in blood pressure (EVBP): Sustained fluctuation in which the difference between maximum and minimum systolic blood pressure values exceeds 20 mmHg.
2. Small variation in blood pressure (SVBP): Sustained fluctuation in which the difference between maximum and minimum systolic blood pressure values ranges between 10 mmHg and 20 mmHg.
3. Hypertensive reaction: Sustained elevation of blood pressure exceeding 130/90 mmHg.
4. Extreme hypertensive reaction: Sustained elevation of blood pressure exceeding 170/120 mmHg.
5. Orthostatic Hypotension (OH): Progressive and sustained decrease in systolic blood pressure of more than 20 mmHg, or in diastolic blood pressure of more than 10 mmHg, or a systolic value below 90 mmHg [2].

### 2.4. Five-Minute ECG Recording in Supine Resting Position

Twelve-lead ECG recordings were obtained using the Cardioscan 12-lead system (D.M.S., Stateline, NV, USA). Commercial software was used to calculate time- and geometry-domain parameters and to conduct spectral analysis of Heart Rate Variability (HRV).

1. Time-domain parameters:
  - SDNN—standard deviation of normal RR intervals
  - rMSSD—root mean square of successive RR interval differences
  - pNN50—percentage of adjacent RR intervals differing by >50 ms
2. Spectral HRV analysis:
  - VLF—very low frequency (0–0.05 Hz)
  - LF—low frequency (0.05–0.17 Hz)
  - HF—high frequency (0.17–0.40 Hz)
  - LF/HF—low-to-high frequency ratio

All short-term ECG recordings were performed under standardized resting conditions, in a quiet, temperature-controlled room. Participants were instructed to breathe calmly and spontaneously, avoiding deep or irregular respiration to minimize respiratory-related variability in spectral HRV indices.

### 2.5. 24-h Holter ECG

Twenty-four-hour ambulatory 12-lead ECG recordings were obtained (Cardioscan, D.M.S., USA). An experienced analyst reviewed and manually corrected beat classifications before further analysis. HRV time- and frequency-domain analyses were performed using the system’s software package.

### 2.6. Statistical Analysis

Data are presented as mean ± standard deviation (SD), median (Mdn) with interquartile range (IQR, 25–75%), or counts (percentage), depending on data type. Normality of continuous variables was assessed using the Smirnov test and by visual inspection of histograms and Q–Q plots.

Group comparisons were performed using parametric tests (Independent Samples *t* test) or nonparametric tests (Chi Square test, Fisher’s exact test, and Mann–Whitney U test) for categorical or non-normally distributed continuous data.

Statistical analyses were conducted using SPSS version 26.0. A two-sided *p* value < 0.05 was considered statistically significant.

## 3. Results

### 3.1. General Findings

Demographic characteristics of the study population are presented in Table 1. As expected, syncope was more prevalent in the syncope groups, while in other groups the frequency of syncope ranged from 30% to 50%. The occurrence of OH during HUTT was higher in the CFS without POTS group compared with both syncope groups.

### 3.2. Syncope Groups

Hemodynamic responses during HUTT in patients with syncope are shown in Table 2. A hypertensive response was significantly more prevalent in the syncope with POTS group. No other significant differences were observed.

Results of the CART battery between the two syncope groups are shown in Table 3. The percentage of normal results in the VM test was significantly higher in the syncope without POTS group. No statistically significant differences were observed for the other tests, nor for overall sympathetic or parasympathetic dysfunction.

Short-term HRV analysis in both syncope groups is shown in Table 4. Mean heart rate values were significantly higher in patients with POTS, which was expected. HF values were significantly lower in patients with POTS.

Parameters from 24 h Holter ECG monitoring are presented in Table 5. The syncope with POTS group had lower average RR intervals over the 24 h monitoring period.

### 3.3. CFS with Insidious Onset Groups

Hemodynamic responses during HUTT in the CFS with insidious onset groups are shown in Table 6. Extreme variation in blood pressure during the passive phase was observed more frequently in the POTS group than in the non-POTS group. No other statistically significant differences were observed.

CART results are shown in Table 7. A normal response during HRB, as well as initial parasympathetic dysfunction, was significantly more frequent in the non-POTS group. Conversely, definite parasympathetic dysfunction was more prevalent in the POTS group.

Short-term HRV parameters in the resting supine position are presented in Table 8. Average heart rate values were significantly higher in the POTS group. In contrast, both pNN50 and rMSSD had lower median values in the POTS group compared to the non-POTS group.

Parameters from 24 h Holter ECG monitoring in the CFS with insidious onset groups are presented in Table 9. No statistically significant differences were observed.

### 3.4. CFS After COVID-19 Groups

Hemodynamic responses in the CFS after COVID-19 groups are presented in Table 10. A significantly higher prevalence of extreme blood pressure variation during the passive phase was observed in the POTS group compared with the non-POTS group. No other statistically significant differences were observed.

CART results are shown in Table 11. Abnormal HRB test results and definite parasympathetic dysfunction were more prevalent in the POTS group, while the non-POTS group showed a higher percentage of initial parasympathetic dysfunction.

Short-term HRV parameters are presented in Table 12. The only statistically significant difference was a higher average heart rate in the POTS group.

Long-term HRV parameters obtained from 24 h Holter ECG monitoring are presented in Table 13. Surprisingly, no statistically significant differences were observed between the two groups.

## 4. Discussion

The prevalence of POTS in the three groups examined in this study ranged from 5% to 7%, depending on the subgroup. These findings are generally consistent with previous studies addressing this topic, particularly those focusing on CFS and POTS. Roerink et al. reported a prevalence of 5.7% among their 419 patients with CFS, a result comparable to our 501 patients with CFS of insidious onset [7,8,9].

Regarding demographics, approximately 70% of patients in all six groups were female. As highlighted in the Introduction, female sex is a well-established risk factor for syncope, POTS, CFS, and PCS [12,13,14,15]. Previous studies have attributed this predisposition to hormonal fluctuations during the menstrual cycle, reduced orthostatic tolerance, and enhanced IgG antibody production in women, particularly in the context of PCS [23,24,25]. Interestingly, although higher syncope prevalence in the syncope groups was expected, 30–50% of patients in the CFS groups also reported a history of syncope. Kenny et al. demonstrated that 21% of patients with vasovagal syncope (VVS) met criteria for CFS, while de Freitas et al. observed syncope in 4.2% of patients following COVID-19 [26,27]. Notably, in our study, 50% of CFS patients without POTS but with a history of syncope developed OH during HUTT, suggesting that this mechanism may underlie syncope and orthostatic intolerance in this subgroup.

In the syncope with POTS group, hypertensive responses during HUTT were significantly more prevalent, observed in 45% of patients. This combined elevation of heart rate and blood pressure suggests a hyperadrenergic mechanism. Petersen et al. reported that young adults with orthostatic hypertension exhibit reduced supine baroreflex sensitivity and decreased cardiac output during tilt, implicating baroreceptor dysfunction as a central mechanism of orthostatic intolerance [28]. Interestingly, although not statistically significant, the non-POTS syncope group exhibited a higher prevalence of positive HUTT results, consistent with the view that POTS alone rarely explains syncope [4].

In both CFS subgroups (insidious onset and post-COVID-19), POTS patients exhibited more frequent extreme blood pressure variations during HUTT. According to Hausenloy et al., large-amplitude oscillations in blood pressure serve as a predictor of VVS during the HUTT [29]. Such fluctuations may represent intermittent sympathetic inhibition with subsequent vagal activation, ultimately leading to sympathetic failure. Moreover, van Campen et al. linked orthostatic intolerance in CFS to reduced cerebral blood flow, even in the absence of overt hemodynamic abnormalities, aligning with Novak’s description of orthostatic cerebral hypoperfusion syndrome [30,31].

In the syncope groups, the non-POTS subgroup exhibited higher rates of normal Valsalva maneuver (VM) results compared to the POTS group. According to Ewing’s criteria, the Valsalva ratio primarily reflects the vagal component of the baroreflex arc, which appeared better preserved in the non-POTS syncope group [32]. The hypertensive response observed in the POTS syncope group may therefore reflect impaired baroreceptor-mediated regulation of short-term blood pressure changes.

Both CFS subgroups with POTS displayed similar CART patterns. In CFS of insidious onset, POTS patients showed marginally higher rates of abnormal VM results and an absence of normal HRB responses compared with non-POTS patients. In CFS post-COVID-19, nearly 91% of POTS patients demonstrated abnormal HRB. While both VM and HRB rely on cardiopulmonary reflexes, their underlying physiology differs: VM primarily reflects baroreceptor function, while HRB depends on the Bainbridge (atrial) reflex. Cui et al. suggested that these reflexes may act simultaneously during deep breathing [33]. The Bainbridge reflex, most pronounced at lower baseline heart rates, may also indirectly engage baroreceptors when heart rates are elevated, as seen in POTS patients [34]. Importantly, definite parasympathetic dysfunction was more prevalent in both CFS with POTS groups, supporting impaired vagal function as a principal mechanism of tachycardia during orthostatic stress.

Short-term HRV analysis in the resting supine position primarily reflects vagal activity [35]. As expected, heart rates were consistently higher in all POTS groups compared with non-POTS groups. In the syncope with POTS group, HF values, representing respiratory-related vagal activity, were lower. Although respiratory sinus arrhythmia contributes to HF, this was not mirrored in HRB differences between syncope groups, suggesting impaired baroreceptor-mediated vagal responses. Similarly, in CFS of insidious onset with POTS, rMSSD and pNN50 values were reduced, consistent with the higher prevalence of definite parasympathetic dysfunction. However, frequency-domain measures are considered more reliable indicators of vagal function in short-term HRV [35]. Unexpectedly, these differences were not observed in CFS post-COVID-19. Shah et al. recently reported that rMSSD measured during 60 s paced breathing distinguished recovered COVID-19 patients from controls, highlighting its diagnostic potential [36]. Jacob et al. likewise demonstrated lower HF, rMSSD, and pNN50 values in POTS compared with non-POTS patients using short-term HRV [37].

Long-term HRV over 24 h predominantly reflects sympathetic modulation due to daily stressors and stimuli [35]. In our study, only the syncope with POTS group exhibited lower HF values, consistent with short-term HRV results, although the difference was not statistically significant. Across all six groups, LF/HF ratios exceeded 2, indicating sympathetic predominance. Such sympathetic overdrive may result in impaired adaptation to stressors, manifesting as fatigue and exercise intolerance, similar to the autonomic imbalance seen in diabetic cardiac autonomic neuropathy [38].

### Study Limitations

Several limitations should be acknowledged. The relatively small sample size (138 patients) represents the main limitation of this study, as it may reduce statistical power and the precision of the estimates. Given the extensive number of parameters analyzed, results were presented primarily using *p*-values to maintain clarity and readability of the tables. Future studies including larger cohorts should aim to provide additional metrics, such as confidence intervals and effect size, to further strengthen the interpretability and robustness of the findings. In addition, since all patients were recruited from a single tertiary care institution, referral bias cannot be excluded. Therefore, the analyzed cohorts may reflect individuals with more severe or complex manifestations of syncope, Chronic Fatigue Syndrome, and post-COVID-19 dysautonomia. Future multicenter studies with larger and more heterogeneous populations are warranted to confirm these findings and improve generalizability. Furthermore, recall bias cannot be excluded, as the classification of CFS and post-COVID-19 CFS patients required confirmation of symptoms lasting longer than six months, which was partly based on patient self-report. Future studies should include prospective symptom tracking or standardized questionnaires to minimize this source of bias.

Second, additional diagnostic measures of autonomic function were not included. Future studies should incorporate continuous blood pressure monitoring to enable the assessment of baroreflex sensitivity, baroreceptor effectiveness index, and blood pressure variability parameters, as these represent important non-invasive markers of sympathetic modulation. Continuous arterial pressure recordings would also allow, in combination with Heart Rate Variability, for the computation of cardiac baroreflex sensitivity, thus providing a more detailed evaluation of baroreflex control. In addition, advanced analytical approaches such as indices of baroreflex regulation, cerebrovascular control, and cardiorespiratory coupling could further enhance the scope of future research, offering a more integrated understanding of autonomic and hemodynamic interactions in POTS. Finally, the inclusion of skin biopsies for the evaluation of epidermal nerve fiber density (ENFD) and sweat gland nerve fiber density (SGNFD) would be valuable for investigating small fiber neuropathy and its potential contribution to autonomic dysfunction in these patients.

Finally, we recommend that future studies adopt a longitudinal design to evaluate changes in autonomic function over time and assess the impact of pharmacological interventions such as beta-blockers or sympathomimetics (e.g., midodrine).

## 5. Conclusions

By employing multiple functional diagnostic approaches to assess autonomic nervous system function, this study characterized a spectrum of dysautonomia in patients with POTS, with distinct patterns depending on the underlying condition (syncope, CFS of insidious onset, or CFS post-COVID-19).

In patients with syncope and POTS during head-up tilt testing, the frequent hypertensive response observed during the passive phase, lower prevalence of normal responses on the Valsalva maneuver (reflecting vagal baroreceptor function), and reduced short-term HRV HF band values suggest that the primary dysfunction in this group is related to impaired baroreceptor function, leading to sympathetic overdrive.

In contrast, in CFS patients with POTS (both insidious onset and post-COVID-19), CART findings demonstrated a higher prevalence of definite parasympathetic dysfunction. Together with frequent extreme blood pressure variations during HUTT, these findings indicate that in CFS, POTS is predominantly related to parasympathetic dysfunction, accompanied by inadequate short-term baroreceptor control of blood pressure.

Overall, delineating dysautonomia profiles in POTS across different disease contexts may provide valuable insight for tailoring therapeutic strategies and improving patient management.

## Figures and Tables

**Table 1:** Demographic characteristic of study population.

	(1) Syncope with POTS N = 20	(2) Syncope Without POTS N = 20	(3) CFS with POTS N = 27	(4) CFS Without POTS N = 27	(5) CFS After COVID-19 with POTS N = 22	(6) CFS After COVID-19 Without POTS N = 22	*p* Value
Age (mean + SD)	38.9 ± 16.6	38.8 ± 16.7	34.6 ± 9.9	34.6 ± 10	33.8 ± 7.9	34 ± 7.7	0.505 ^a^
Female (n, %)	14 (70%)	14 (70%)	18 (67.7%)	18 (67.7%)	15 (68.2%)	15 (68.2%)	1 ^b^
Previous Syncope (n, %)	20 ^3,4,5,6^ (100%)	20 ^3,4,5,6^ (100%)	8 ^1,2^ (29.6%)	10 ^1,2^ (37%)	7 ^1,2^ (31.8%)	11 ^1,2^ (50%)	<0.001 ^b^
Syncope+, OH+ (n,% of patient with prev. syncope)	0 ^4^^,6^	0 ^4^	0 ^4^	5 ^1,2,^^3,5^ (50%)	0 ^4^	3 ^1,2^ (27.3%)	<0.001 ^b^
Syncope-, OH+ (n, %)	0	0	0	0	0	1 (4.5%)	0.609 ^c^

POTS—Postural Tachycardia Syndrome; CFS—Chronic Fatigue Syndrome (groups 3. and 4. refer to CFS with insidious onset); HUTT—Head-up tilt test; SD—standard deviation; Syncope+, OH+—Patients with previous syncope with Orthostatic Hypotension during head-up tilt test; Syncope-, OH+—Patients without previous syncope with Orthostatic Hypotension during head-up tilt test; ^a^—Pearson Chi Square; ^b^—Fisher Exact Test; ^c^—One way ANOVA; Superscript—significant difference between examined group and group named by superscript; Underline—significant difference between groups with Bonferroni correction (*p* < 0.003).

**Table 2:** Responses during HUTT in Syncope groups.

	Syncope with POTS N = 20	Syncope Without POTS N = 20	*p* Value
Positive HUTT (n, %)	9 (45%)	13 (65%)	0.204 ^a^
EVBP (n, %)	12 (60%)	9 (45%)	0.342 ^a^
SVBP (n, %)	0	3 (15%)	0.072 ^a^
Hypertensive response (n, %)	9 (45%)	1 (5%)	0.003 ^a^
Extreme Hypertensive response (n, %)	4 (20%)	0	0.106 ^b^

POTS—Postural Tachycardia Syndrome; HUTT—Head-up tilt test; EVBP—Extreme variation in blood pressure; SVBP—Small variation in blood pressure; ^a^—Pearson Chi Square; ^b^—Fisher Exact Test.

**Table 3:** Results of Cardiovascular reflex tests in Syncope groups.

	Syncope with POTS N = 20	Syncope Without POTS N = 20	*p* Value
Abnormal HGT (n, %)	19 (95%)	19 (95%)	1 ^a^
Abnormal OH (n, %)	0	0	/
Normal VM (n, %)	6 (30%)	14 (70%)	0.011 ^b^
Abnormal VM (n, %)	9 (45%)	4 (20%)	0.091 ^b^
Abnormal HRB (n, %)	13 (65%)	12 (60%)	0.744 ^b^
Abnormal HRS (n, %)	20 (100%)	18 (90%)	0.487 ^a^
Sympathetic Dysfunction (n, %)	19 (95%)	19 (95%)	1 ^a^
Definite Parasympathetic Dysfunction (n, %)	16 (80%)	11 (55%)	0.091 ^b^
Scor of AN (n, %)	6.45 ± 1.39	5.6 ± 1.6	0.448 ^c^

POTS—Postural Tachycardia Syndrome; HGT—Hand grip test; OH—Orthostatic Hypotension; VM—Valsalva maneuver; HRB—Heart rate response to deep breathing; HRS—Heart rate response to standing; AN—Autonomic Neuropathy; ^a^—Pearson Chi Square; ^b^—Fisher Exact Test; ^c^—Independent *t* test.

**Table 4:** Short-term Hear Rate Variability results in Syncope groups.

	Syncope with POTS N = 20	Syncope Without POTS N = 20	*p* Value
HR (bpm) (mean +SD)	90.05 ± 14.18	75.75 ± 15.65	0.005 ^a^
SDNN (ms) (Mdn + IQR)	47 (37–98)	60 (45–107)	0.470 ^b^
rMSSD (ms) (Mdn + IQR)	23.5 (14-34-34.75)	42 (23–90)	0.057 ^b^
pNN50 (%) (Mdn + IQR)	1 (0–11.5)	8 (0–27)	0.169 ^b^
VLF (ms^2^) (Mdn + IQR)	823.5 (396.35–2573.1)	374.9 (151.2–961.1)	0.057 ^b^
LF (ms^2^) (Mdn + IQR)	323.3 (153.75–588.05)	386.1 (142.5–728.1)	0.684 ^b^
HF (ms^2^) (Mdn + IQR)	112.3 (54.85–247.95)	221.8 (116.8–446.9)	0.042 ^b^
LF/HF (Mdn + IQR)	1.9 (1.15–6.9)	1.6 (0.7–3.9)	0.196 ^b^

POTS—Postural Tachycardia Syndrome; HR—Heart rate; bpm—beat per minute; SDNN—standard deviation of normal RR intervals; rMSSD—the square root of the mean of the squares of the successive differences between adjacent normal RR intervals; pNN50—the percentage of intervals > 50 ms different from preceding interval; VLF—very low frequency component of HRV (0–0.05 Hz); LF—low frequency component of HRV (0.05–0.17 Hz); HF—high frequency component of HRV (0.17–0.4 Hz); LF/HF—low frequency/high frequency ratio of HRV; SD—standard deviation; Mdn—Median; IQR—Interquartile range (25–75%); ^a^—One Way Anova; ^b^—Mann–Whitney U Test.

**Table 5:** Long-term Heart Rate Variability parameters in syncope groups.

	Syncope with POTS N = 20	Syncope Without POTS N = 20	*p* Value
Average RR (ms) (mean + SD)	722.24 ± 55.79	836.4 ± 129.36	0.023 ^a^
HR (bmp) (mean + SD)	79.78 ± 8.54	72 ± 9.21	0.063 ^a^
SDNN (ms) (mean + SD)	148.89 ± 33.83	171.5 ± 50.55	0.261 ^a^
rMSSD (ms) (mean + SD)	44.56 ± 27.14	64.08 ± 43.96	0.256 ^a^
pNN50 (%) (mean + SD)	12.44 ± 8.82	17.58 ±14.37	0.357 ^a^
VLF (ms^2^) (Mdn + IQR)	2192.8 (1854.75–3222.95)	2218.1 (936.75–4066.8)	0.808 ^b^
LF (ms^2^) (Mdn + IQR)	939 (577–1412.9)	795.4 (343.18–1450.5)	0.917 ^b^
HF (ms^2^) (Mdn + IQR)	270.9 (222.3–537.4)	395 (86.18–644.35)	0.702 ^b^
LF/HF (Mdn + IQR)	3.09 (2.1–3.55)	2.52 (2.18–3.23)	0.554 ^b^

POTS—Postural Tachycardia Syndrome; HR—Heart rate; bpm—beat per minute; SDNN—standard deviation of normal RR intervals; rMSSD—the square root of the mean of the squares of the successive differences between adjacent normal RR intervals; pNN50—the percentage of intervals > 50 ms different from preceding interval; VLF—very low frequency component of HRV (0–0.05 Hz); LF—low frequency component of HRV (0.05–0.17 Hz); HF—high frequency component of HRV (0.17–0.4 Hz); LF/HF—low frequency/high frequency ratio of HRV; SD—standard deviation; Mdn—Median; IQR—Interquartile range (25–75%); ^a^—One Way Anova; ^b^—Mann–Whitney U Test.

**Table 6:** Responses during head-up tilt test in CFS with insidious onset groups.

	CFS with POTS N = 27	CFS Without POTS N = 27	*p* Value
Positive HUTT (n, %)	5 (18.5%)	9 (33.3%)	0.214 ^a^
EVBP (n, %)	20 (74.1%)	12 (44.4%)	0.027 ^a^
SVBP (n, %)	4 (14.8%)	5 (18.5%)	1 ^b^
Hypertensive response (n, %)	7 (25.9%)	9 (33.3%)	0.551 ^a^
Extreme Hypertensive response (n, %)	4 (14.8%)	3 (11.1%)	1 ^b^

POTS—Postural Tachycardia Syndrome; CFS—Chronic Fatigue Syndrome with insidious onset; HUTT—Head-up tilt test; EVBP—Extreme variation in blood pressure; SVBP—Small variation of blood pressure; ^a^—Pearson Chi Square; ^b^—Fisher Exact Test.

**Table 7:** Results of Cardiovascular reflex tests in CFS with insidious onset groups.

	CFS with POTS N = 27	CFS with POTS N = 27	*p* Value
Abnormal HGT (n, %)	26 (96.3%)	25 (92.6%)	1 ^a^
Abnormal OH (n, %)	0	2 (7.4%)	0.491 ^a^
Abnormal VM (n, %)	9 (33%)	3 (11.1%)	0.05 ^b^
Normal HRB (n, %)	0	8 (29.6%)	0.004 ^a^
Abnormal HRB (n, %)	22 (81.5%)	16 (59.3%)	0.074 ^b^
Abnormal HRS (n, %)	24 (88.9%)	26 (96.3%)	0.610 ^a^
Initial Parasympathetic Dysfunction (n, %)	3 (11.1%)	11 (40.7%)	0.013 ^b^
Definite Parasympathetic Dysfunction (n, %)	23 (85.2%)	16 (59.3%)	0.033 ^b^
Scor of AN (n, %)	6.52 ± 1.05	5.85 ± 1.56	0.071 ^c^

POTS—Postural Tachycardia Syndrome; CFS—Chronic Fatigue Syndrome with insidious onset; HGT—Hand grip test; OH—Orthostatic Hypotension; VM—Valsalva maneuver; HRB—Heart rate response to deep breathing; HRS—Heart rate response to standing; AN—Autonomic Neuropathy. ^a^—Fisher Exact Test, ^b^—Pearson Chi Square, ^c^—Independent *t* test.

**Table 8:** Short-term Heart Rate Variability in CFS with insidious onset groups.

	CFS with POTS N = 27	CFS Without POTS N = 27	*p* Value
HR (bpm) (mean +SD)	90.63 ± 13.25	74.46 ± 10.53	<0.001 ^a^
SDNN (ms) (Mdn + IQR)	61 (30.5–81)	48 (72.5–144.25)	0.082 ^b^
rMSSD (ms) (Mdn + IQR)	26 (15.5–81)	32 (62.5–106.5)	0.02 ^b^
pNN50 (%) (Mdn + IQR)	3 (0–6.5)	11.5 (2.75–22.75)	0.01 ^b^
VLF (ms^2^) (Mdn + IQR)	550.2 (172.45–1414.45)	515.25 (244.95–1396.25)	0.649 ^b^
LF (ms^2^) (Mdn + IQR)	440.3 (211.65–739.1)	431.9 (268.9–647.33)	0.973 ^b^
HF (ms^2^) (Mdn + IQR)	106.2 (51.75–266.05)	147.15 (78.575–408.5)	0.255 ^b^
LF/HF (Mdn + IQR)	3.6 (1.65–5.85)	2.2 (1.15–3.2)	0.067 ^b^

POTS—Postural Tachycardia Syndrome; CFS—Chronic Fatigue Syndrome with insidious onset; HR—Heart rate; bpm—beat per minute; SDNN—standard deviation of normal RR intervals; rMSSD—the square root of the mean of the squares of the successive differences between adjacent normal RR intervals; pNN50—the percentage of intervals > 50 ms different from preceding interval; VLF—very low frequency component of HRV (0–0.05 Hz); LF—low frequency component of HRV (0.05–0.17 Hz); HF—high frequency component of HRV (0.17–0.4 Hz); LF/HF—low frequency/high frequency ratio of HRV; SD—standard deviation; Mdn—Median; IQR—Interquartile range (25–75%); ^a^—One Way Anova; ^b^—Mann–Whitney U Test.

**Table 9:** Long-term Heart Rate Variability and Blood pressure parameters in CFS with insidious onset group.

	CFS with POTS N = 27	CFS Without POTS N = 27	*p* Value
Average RR (ms) (Mdn + IQR)	745.7 (669.5–808)	783.5 (680–834)	0.479 ^a^
HR (bmp) (Mdn + IQR)	79 (73–88)	76 (71–87)	0.479 ^a^
SDNN (ms) (Mdn + IQR)	156 (119–168)	128 (114–164)	0.659 ^a^
rMSSD (ms) (Mdn + IQR)	30 (24–32)	35 (24–42)	0.596 ^a^
pNN50 (%)(Mdn + IQR)	7 (4–11)	7 (4–16)	0.791 ^a^
VLF (ms^2^) (Mdn + IQR)	2192.8 (1854.75–3222.95)	2218.1 (936.75–4066.8)	0.515 ^a^
LF (ms^2^) (Mdn + IQR)	971.6 (746.25–1477.58)	683.5 (530.93–1124.55)	0.237 ^a^
HF (ms^2^) (Mdn + IQR)	287.35 (167.6–541.8)	264.55 (187.15–416)	0.897 ^a^
LF/HF (Mdn + IQR)	2.85 (1.8–6)	2.85 (1.92–4.25)	0.696 ^a^

POTS—Postural Tachycardia Syndrome; CFS—Chronic Fatigue Syndrome with insidious onset; HR—Heart rate; bpm—beat per minute; SDNN—standard deviation of normal RR intervals; rMSSD—the square root of the mean of the squares of the successive differences between adjacent normal RR intervals; pNN50—the percentage of intervals > 50 ms different from preceding interval; VLF—very low frequency component of HRV (0–0.05 Hz); LF—low frequency component of HRV (0.05–0.17 Hz); HF—high frequency component of HRV (0.17–0.4 Hz); LF/HF—low frequency/high frequency ratio of HRV; Mdn—Median; IQR—Interquartile range (25–75%); ^a^—Mann–Whitney U Test.

**Table 10:** Responses during HUTT in CFS after COVID-19 groups.

	CFS After COVID-19 with POTS N = 22	CFS After COVID-19 Without POTS N = 22	*p* Value
Positive HUTT (n, %)	4 (18.2%)	10 (45.5%)	0.052 ^a^
EVBP (n, %)	19 (86.4%)	12 (54.5%)	0.021 ^a^
SVBP (n, %)	0	2 (9.1%)	0.488 ^b^
Hypertensive response (n, %)	8 (36.4%)	4 (18.2%)	0.176 ^a^
Extreme Hypertensive response (n, %)	5 (22.7%)	3 (13.6%)	0.698 ^b^

POTS—Postural Tachycardia Syndrome; CFS—Chronic Fatigue Syndrome; HUTT—Head-up tilt tet; EVBP—Extreme variation in blood pressure; SVBP—Small variation of blood pressure; ^a^—Pearson Chi Square; ^b^—Fisher Exact Test.

**Table 11:** Results of Cardiovascular reflex tests in CFS after COVID-19 groups.

	CFS After COVID-19 with POTS N = 22	CFS After COVID-19 with POTS N = 22	*p* Value
Abnormal HGT (n, %)	21 (95.5%)	22 (100%)	1 ^a^
Abnormal OH (n, %)	0	2 (9.1%)	0.488 ^a^
Abnormal VM (n, %)	36.4 (33%)	11 (50%)	0.361 ^b^
Abnormal HRB (n, %)	20 (90.9%)	10 (45.5%)	0.001 ^b^
Abnormal HRS (n, %)	21 (95.5%)	18 (81.8%)	0.345 ^a^
Initial Parasympathetic Dysfunction (n, %)	1 (4.5%)	7 (31.8%)	0.046 ^b^
Definite Parasympathetic Dysfunction (n, %)	21 (95.5%)	15 (68.2%)	0.046 ^b^
Scor of AN (n, %)	6.64 ± 1	6.59 ± 1.33	0.889 ^c^

POTS—Postural Tachycardia Syndrome; CFS—Chronic Fatigue Syndrome; HGT—Hand grip test; OH—Orthostatic Hypotension; VM—Valsalva maneuver; HRB—Heart rate response to deep breathing; HRS—Heart rate response to standing; AN—Autonomic Neuropathy; ^a^—Fisher Exact Test; ^b^—Pearson Chi Square; ^c^—Independent *t* test.

**Table 12:** Short-term Heart Rate Variability in CFS after COVID-19 groups.

	CFS After COVID-19 with POTS N = 22	CFS After COVID-19 Without POTS N = 22	*p* Value
HR (bpm) (mean + SD)	94 ± 15.01	74.4 ± 10.64	0.001 ^a^
SDNN (ms) (mean + SD)	60.81 ± 33.98	89.47 ± 46.74	0.098 ^a^
rMSSD (ms) (Mdn + IQR)	65 (20–85)	71 (24–106)	0.474 ^b^
pNN50 (%) (Mdn + IQR)	2 (0–5)	5 (2–20)	0.237 ^b^
VLF (ms^2^) (Mdn + IQR)	262.1 (199.9–544.4)	590.5 (249–726)	0.164 ^b^
LF (ms^2^) (Mdn + IQR)	206.8 (71.7–528.3)	275.5 (215.3–524)	0.305 ^b^
HF (ms^2^) (Mdn + IQR)	101.5 (32.3–137.6)	161.3 (62.6–453.6)	0.217 ^b^
LF/HF (Mdn + IQR)	2.2 (1.8–2.6)	1.9 (0.6–3)	0.646 ^b^

POTS—Postural Tachycardia Syndrome; CFS—Chronic Fatigue Syndrome; HR—Heart rate; bpm—beat per minute; SDNN—standard deviation of normal RR intervals; rMSSD—the square root of the mean of the squares of the successive differences between adjacent normal RR intervals; pNN50—the percentage of intervals > 50 ms different from preceding interval; VLF—very low frequency component of HRV (0–0.05 Hz); LF—low frequency component of HRV (0.05–0.17 Hz); HF—high frequency component of HRV (0.17–0.4 Hz); LF/HF—low frequency/high frequency ratio of HRV; SD—standard deviation; Mdn—Median; IQR—Interquartile range (25–75%); ^a^—One Way Anova; ^b^—Mann–Whitney U Test.

**Table 13:** Long-term Heart Rate Variability and Blood pressure parameters in CFS with insidious onset after COVID-19 group.

	CFS After COVID-19 with POTS N = 27	CFS After COVID-19 Without POTS N = 27	*p* Value
Average RR (ms) (Mdn + IQR)	755.6 (676.1–782.9)	720 (685.73–760.65)	0.724 ^a^
HR (bmp) (Mdn + IQR)	78 (75–87.5)	82.5 (78.25–86)	0.724 ^a^
SDNN (ms) (Mdn + IQR)	196 (123.5–209)	120 (108.5–147.5)	0.127 ^a^
rMSSD (ms) (Mdn + IQR)	37 (27–41)	31 (25.2–55)	0.524 ^a^
pNN50 (%)(Mdn + IQR)	11 (7–15.5)	7 (5–9.5)	0.222 ^a^
VLF (ms^2^) (Mdn + IQR)	2629.1 (1381.15–3315.8)	1702.5 (1196.88–2056.15)	0.354 ^a^
LF (ms^2^) (Mdn + IQR)	983.3 (545.55–1272)	739.1 (579.25–869.28)	0.435 ^a^
HF (ms^2^) (Mdn + IQR)	296.1 (204.12–405.05)	256.05 (154.5–530.25)	0.833 ^a^
LF/HF (Mdn + IQR)	3 (2.2–4.35)	2.65 (1.55–2.78)	0.284 ^a^

POTS—Postural Tachycardia Syndrome; HR—Heart rate; bpm—beat per minute; SDNN—standard deviation of normal RR intervals; rMSSD—the square root of the mean of the squares of the successive differences between adjacent normal RR intervals; pNN50—the percentage of intervals > 50 ms different from preceding interval; VLF—very low frequency component of HRV (0–0.05 Hz); LF—low frequency component of HRV (0.05–0.17 Hz); HF—high frequency component of HRV (0.17–0.4 Hz); LF/HF—low frequency/high frequency ratio of HRV; Mdn—Median; IQR—Interquartile range (25–75%); ^a^—Mann–Whitney U Test.

## Data Availability

Available upon reasonable request due to privacy reasons.
